# Predicting executive functioning from walking features in Parkinson’s disease using machine learning

**DOI:** 10.1038/s41598-024-80144-4

**Published:** 2024-11-27

**Authors:** Artur Piet, Johanna Geritz, Pascal Garcia, Mona Irsfeld, Frédéric Li, Xinyu Huang, Muhammad Tausif Irshad, Julius Welzel, Clint Hansen, Walter Maetzler, Marcin Grzegorzek, Nico Bunzeck

**Affiliations:** 1 Institute of Medical Informatics, University of Luebeck, Ratzeburger Allee 160, 23562 Luebeck, Germany; 2 Department of Neurology, University Hospital Schleswig-Holstein, Kiel, Germany; 3 Department of Psychology and Center of Brain, Behavior and Metabolism (CBBM), University of Luebeck, Luebeck, Germany; 4 German Research Center for Artificial Intelligence, Luebeck, Germany

**Keywords:** Executive functioning, Parkinson’s disease, Machine learning, Walking features, Human behaviour, Neurological disorders

## Abstract

Parkinson’s disease is characterized by motor and cognitive deficits. While previous work suggests a relationship between both, direct empirical evidence is scarce or inconclusive. Therefore, we examined the relationship between walking features and executive functioning in patients with Parkinson’s disease using state-of-the-art machine learning approaches. A dataset of 103 geriatric Parkinson inpatients, who performed four walking conditions with varying difficulty levels depending on single task walking and additional motor and cognitive demands, was analyzed. Walking features were quantified using an inertial measurement unit (IMU) system positioned at the patient’s lower back. The analyses included five imputation methods and four regression approaches to predict executive functioning, as measured using the Trail-Making Test (TMT). Multiple imputation by chained equations (MICE) in combination with support vector regression (SVR) reduce the mean absolute error by about 4.95% compared to baseline. Importantly, predictions solely based on walking features obtained with support vector regression mildly but significantly correlated with Δ−TMT values. Specifically, this effect was primarily driven by step time variability, double limb support time variability, and gait speed in the dual task condition with cognitive demands. Taken together, our data provide direct evidence for a link between executive functioning and specific walking features in Parkinson’s disease.

**Supplementary Information:**

The online version contains supplementary material available at 10.1038/s41598-024-80144-4.

## Introduction

Idiopathic Parkinson’s disease is characterized by both motor and cognitive deficits^1^, which may have a close connection. For instance, walking impairments, including slow walking speed and gait variability, could be related to cognitive abilities^2,3^. Importantly, frequently observed deficits in executive functioning^4,5^, which constitutes a set of abilities, including inhibition and inference control, working memory and cognitive flexibility^6^, were associated with reduced gait speed^7,8^. While structural and functional impairments within fronto-striatal and limbic circuits offer a physiological basis^1,8,9^, direct evidence for a link between deficits in executive functioning and specific markers of motor abilities in Parkinson’s disease is scarce.

Spatio-temporal walking features are manifold and their relationship with cognitive abilities appears to change with disease progression depending on task demands. For example, in dual task conditions gait speed decreased in healthy older adults and patients with Parkinson’s disease, but gait variability only increased in Parkinson’s disease^10,11^. Importantly, executive functioning in Parkinson’s disease was significantly correlated with gait variability in a dual task condition but not during single task walking^12^. Together with other studies, also indicating reduced executive functioning in combination with impaired walking abilities in Parkinson’s disease^5^, this suggests a strategic adaptation to task demands in healthy older adults, which breaks down in patients with Parkinson’s disease.

Apart from gait speed and gait variability, previous studies have focused on stride length and postural control, which might all have a unique relationship with impaired executive functioning in Parkinson’s disease^13–15^. However, not all studies come to the same conclusion^13,16–18^, which may be due to differences and limitations in methodological approaches and the fact that different walking conditions (e.g. comfortable vs. fast) have been used in combination with different cognitive challenges (e.g. single vs. dual task). Along these lines, recent advances in machine learning offer new ways to analyze complex data to gain new insights into such issues^19^. For instance, decision tree classification of sensor-based walking features, together with non-walking features, have been used to classify healthy controls vs. patients with Parkinson’s disease, as well as their disease stage^20^. Furthermore, motor deficits in Parkinson’s disease (i.e. MDS-UPDRS scores) could be predicted on the basis of a set of non-walking features using deep neural network (DNN) analysis^21^.

The overall goal of this study is to provide evidence for a relationship between executive functioning, as assessed with the Trail-Making Test (TMT, see methods), and spatio-temporal walking features, assessed with inertial measurement unit (IMU) wearable sensors, in a sample of 103 acutely hospitalized patients with Parkinson’s disease (Table 1). For the gait analysis, participants were asked to walk a marked straight distance of 20 m four times under four walking conditions. In condition one (single task “ST” normal pace), the distance was to be covered at a self-selected comfortable gait speed. In condition two (ST fast pace), participants were asked to walk as fast as possible without running. In condition three (dual task “DT” walking-cognitive), participants were asked to consecutively subtract seven from a given three-digit number as fast as possible while walking at a fast pace. In condition four (DT walking-motor), predetermined boxes on a paper sheet had to be crossed with a pencil as quickly as possible while walking at a fast pace.

**Table 1:** Demographic and clinical parameters of the patients (*n* = 103 that were entered into the analysis), also referred to as meta features.

Meta feature	Avg.	SD	Range	Missingness
Age [years]	72.62	8.10	[48–87]	0.0%
Disease duration [years]	9.74	6.76	[0–25]	18.2%
Education [years]	10.49	2.07	[6–19]	0.9%
BMI [kg/m²]	26.42	4.63	[16–48]	8.3%
LEDD [mg]	785.22	368.93	[100-1811]	16.0%
MoCA [points]	23.20	3.37	[15-29]	1.9%
Walking aid usage [%]	17.05			18.8%
MDS-UPDRS III total	29.68	15.07	[4–61]	11.7%
DIA-S total	2.33	2.41	[0–9]	6.8%
Female [%]	31.08			0.0%
Hoehn and Yahr scale	3.13	0.89	[0–4]	6.8%

Abbreviations: Avg., Average; SD, standard deviation; BMI, Body Mass Index; DIA-S, *Depression im Alter* Scale; LEDD, levodopa equivalence daily dose (in milligram, mg); MoCA, Montreal Cognitive Assessment total score. Walking aid usage (%) reflects the entire sample and indicates whether each individual did or did not use a walking aid.

We hypothesized an accurate prediction of executive functioning on the basis of walking and non-walking features combined (analysis 1), and on the basis of walking features alone (analysis 2, Table S1). To this end, we explored the suitability of different machine learning approaches for data imputation and regression. Data imputation was first applied to fill in missing data values to standardize the inputs of machine learning models. In a second step, machine learning models for regression were trained on the imputed data to predict the patients’ executive functioning.

## Methods

### Data acquisition

Data were collected at the Department of Neurology, University Hospital Schleswig-Holstein Campus Kiel (Germany), between 2017 and 2021 as part of the ComOn study^13^. All patients with Parkinson’s disease were diagnosed according to the Movement Disorder Society (MDS) clinical diagnostic criteria by the attending neurologist. The main reasons for hospitalization were deterioration in mobility or general condition, recent falls or medication adjustment due to reduced drug effects. Patients were included in this study when they were older than 50 years, capable of walking at least 20 m with or without a walking aid. They were excluded with a score lower than five on the Montreal Cognitive Assessment (MoCA)^22,23^ indicating severe dementia or if they reported more than two falls in the past week.

Verbal and written informed consent was obtained from all participants and, if necessary, their legal representative or assistant (e.g., in case of cognitive deficits or dementia). Detailed information regarding further inclusion criteria as well as the complete study protocol were already published elsewhere^24^. The ComOn study, including the experimental protocols reported here, was reviewed by the ethics committee of the Medical Faculty of the University of Kiel (ethics application number D 427/17). For more information about the subjects’ demographics and the assessments on motor and cognitive functions, also referred to as meta features, see below (and Table 1). All methods were performed in accordance with the relevant guidelines and regulations.

Executive functioning was assessed using the Trail-Making Test (TMT^25–27^), which consists of two parts, referred to as TMT-A and TMT-B. TMT-A involves connecting a series of numbered circles (1–25) randomly distributed on a sheet of paper in numerical order as quickly as possible. TMT-B involves connecting a series of circles that alternate between numbers and letters (1–13 and A-L) in an ascending order (e.g., 1-A-2-B-3-C, etc.) as quickly and accurately as possible. Both parts are scored by time of completion in seconds. While the TMT-A has been predominantly linked to psycho-motor speed, the TMT-B demands higher cognitive processes, such as cognitive flexibility and working memory, as parts of executive functioning^28^. Following standardized test instructions, participants were made aware of mistakes. To better account for individual executive functioning-related processing speed, the time difference between TMT-B and TMT-A was defined as Δ-TMT. As such, Δ-TMT provides a suitable measure of executive functioning by disentangling the motor speed component of TMT-A from the more complex TMT-B^29^. Note that the TMT is one proxy, out of many, for executive functioning^30^. See discussion.

All data were taken from the ComOn dataset (see above), which, at this time, included *n* = 200 patients with Parkinson’s disease; however, Δ-TMT scores were not provided for *n* = 72 patients, and could, therefore, not be included here. The number of occurrences and the distribution of Δ-TMT scores in the dataset are depicted in the supplementary material (Figure S1), ranging from − 5.0 to 340.0 s, with an average of 125.2 s and a standard-deviation (SD) of 76.4 s.

As described in our previous work^13,31^, walking features were quantified using a RehaGait^®^ (Hasomed, Magdeburg, Germany^32^) IMU system. It is CE-certified and includes a triaxial accelerometer (± 16 g) and a triaxial gyroscope (± 2,000/s). The sensor was attached to the patient’s lower back at the level of the fifth lumbar vertebra before the gait assessment. Data were collected at a sampling frequency of 100 Hz and transmitted during the measurement via Bluetooth to a tablet with the RehaGait^®^ application modified for the ComOn study in cooperation with the manufacturer. The raw data were processed later to extract spatio-temporal walking features using a validated algorithm for step detection in patients with Parkinson’s disease^33^. Additionally, a linear correction was performed to account for the initial acceleration of each walking feature (except number of steps and gait speed), normalizing for gait speed (1 m/s) as recommended in previous biomechanical studies on sensor-based walking features^34^.

For the gait analysis, participants were asked to walk a marked straight distance of 20 m four times under four walking conditions (see introduction): single task “ST” normal pace, ST fast pace, dual task “DT” walking-cognitive, and DT walking-motor. The DT walking-motor condition was only possible for patients without a walking aid. Walking conditions were performed in the following order if patients had the capacity: ST fast pace, ST normal pace, DT walking-motor, DT walking-cognitive. A randomization of the tasks was not feasible for practical reasons due to the embedding in the comprehensive movement analysis protocol of the ComOn study^24^.

### Feature extraction and preprocessing

From the raw IMU records, the following walking features were computed to characterize different domains of the gait profile^14^ (Table S1): number of steps, gait speed, time, step time, stride time, stance time, swing time, asymmetry, step time variability (STV), double limb support time (DLS), and double limb support time variability (DLSV). For each walking condition, subjects with more than 75% of missing data values were excluded. Additionally, for gait speed 14 outlier values (defined as ± 3SD values from the mean) were removed. This reduced the total number of data instances (i.e. number of examples in the dataset) to *n* = 325, resulting in 102 remaining patients with ST normal pace, 82 patients with ST fast pace, 73 patients with DT walking-cognitive and 68 patients with DT walking-motor. In total, we used the data from 103 different patients in all four walking conditions.

### Feature imputation

The percentage of missing values in the dataset was 14.21%, with each individual walking condition accounting for ST normal 12.66%, ST fast 11.81%, DT walking-cognitive 12.62%, and DT walking-motor 21.38%, respectively. Thus, we used the following imputation methods taken from the literature^35–37^: K-Nearest Neighbors algorithm (KNN), Multiple Imputation by Chained Equations (MICE)^38^, Miss Forest (MF)^39^, and Multiple Imputation with Denoising Autoencoders (MIDAS)^40^.

The imputation techniques were compared to a baseline, in which missing values were replaced by the mean values of each feature. Regression evaluation metrics (mean absolute error [MAE], root mean square error [RMSE] and Pearson’s correlation) were then obtained after training one of the four regression methods (see 2.5). This comparison was carried out for each of the four walking conditions separately.

### Feature selection

To remove unnecessary or redundant features and to reduce the complexity of our classification model, we applied feature selection. Feature selection can improve the performance of a model and determine the interdependence between features and the prediction variable^41^. Feature ranking, which quantifies the capability of a feature to predict the desired variable (here Δ-TMT score), is a common approach to feature selection. This was done using recursive feature elimination (RFE). RFE identifies the feature with the least importance for the regression. Subsequently, this feature is removed. This process is repeated iteratively, eliminating features one by one. The result is a ranking of feature importance based on the reduction in impurity of the splits, known as Gini importance^42^. All Gini import values were obtained using a random forest and were optimized to minimize the mean absolute error (MAE, see evaluation) between Δ-TMT prediction and true Δ-TMT score. The best feature sets selected by RFE in all tested configurations (i.e. for all walking conditions, using either meta + walking features or walking features only) are provided in Table S2. See also Figure S4 and S5, respectively, for more details.

### Regression

For the regression analysis, we aimed to find the best performing combination of imputation technique (to fill in missing values) and regression model (to predict the outcome variable, i.e. here Δ-TMT). We used commonly employed machine learning approaches such as random forest (RF), support vector regression (SVR), extreme gradient boosting (XGB), and multilayer perceptron (MLP). Each of these models has its own set of hyperparameters that can significantly impact their performance. All hyperparameters of our models were tuned using Bayesian optimization^43^. Bayesian optimization is a hyperparameter tuning technique that combines prior knowledge and observed results to guide the search for optimal hyperparameter values. It iteratively explores the hyperparameter space, evaluating different combinations of hyperparameters and updating its beliefs about which settings are likely to yield the best performance. The combination of hyperparameter values resulting in the lowest MAE was chosen in each tested configuration.

### Evaluation

Regression models are typically assessed by comparing the trained model predictions with the actual (observed) data^44^. Here, we used two standard metrics, the MAE and the root mean square error (RMSE) to evaluate the prediction errors of the regression models, and indirectly the performance of the imputation methods. The lower the error, the higher the performance of the model. MAE is robust to outliers and can be interpreted directly since the error is expressed in units of the outcome variable (here Δ-TMT scores in seconds). In contrast, the RMSE is the square root of the average of squared errors, penalizing outliers, i.e., larger errors. It is also defined as the standard deviation of the residuals, i.e., prediction error = (y_true_ - y_estimated_). A third evaluation metric is the correlation between the predicted and actual Δ-TMT scores using the Pearson correlation coefficient r.

The evaluation was performed using a leave-one-out cross-validation to provide evaluation performances that are realistic regarding the capacity of the trained models to generalize to unseen subjects.

## Results

### Imputation

As shown in Table 2, MICE in combination with SVR yielded the best performance, across all four walking conditions for the MAE, RMSE and r, respectively. Table 3 shows the best performing combination of imputation and regression methods using only the walking features, with MICE combined with SVR also returning the best results across all methods we compared. More details about the performance of all tested combinations of imputation and regression methods using only the walking features for each of the four walking conditions can be found in supplementary material Figure S2 and S3. Figure S6 shows a comparison of the ground truth to the Δ-TMT values predicted using the meta and walking features, and Figure S7 only the walking features. Importantly, a significant correlation between Δ-TMT and walking features was only observed in the DT walking-cognitive condition (Table 3). Note that r-values in Table 2 represent the slopes of the trend lines for Figure S6, and r-values in Table 3 represent the slopes of the trend lines for Figure S7.

**Table 2:** Regression results for all four walking conditions after imputing missing values using SVR using both feature types (walking and meta features). The p-values are computed between the estimations of the SVR model and the Δ-TMT values.

Walking conditions	Methods	MAE	RMSE	Pearson’s *r* [*p*-value]
ST normal pace	MICE + SVR	47.24	66.98	0.46 [< 0.00001]
ST fast pace	MICE + SVR	51.88	71.34	0.44 [< 0.00002]
DT walking-motor	MICE + SVR	46.94	68.22	0.45 [< 0.00005]
DT walking-cognitive	MICE + SVR	**44.09**	**66.73**	**0.47 [< 0.00001]**

**Table 3:** Regression results for all four walking conditions after imputing missing values using SVR using only walking features. The p-values are computed between the estimations of the SVR model and the Δ-TMT values.

Walking conditions	Methods	MAE	RMSE	Pearson’s *r* [*p*-value]
ST normal pace	MICE + SVR	54.48	72.47	0.07 [< 0.5]
ST fast pace	MICE + SVR	58.20	75.80	0.03 [< 0.74]
DT walking-motor	MICE + SVR	54.65	72.78	0.12 [< 0.3]
DT walking-cognitive	MICE + SVR	**49.14**	**69.50**	**0.31 [< 0.007]**

A specific quantification of the improvement in MAE by the MICE imputation in combination with SVR compared to the baseline revealed the following: for the DT walking-cognitive condition, in the analysis with both meta and walking features (Figure S2), there was an improvement of 5.49% (i.e., MAE of 46.65 was reduced to 44.09). For the DT walking-cognitive condition, in the analysis with walking features alone (Figure S3), there was an improvement of 4.95% (i.e., MAE of 51.70 was reduced to 49.14). The relative MAE^45^, defined as MAE_1_ divided by MAE_2_, where MAE_1_ is MICE + SVR and MAE_2_ is the baseline, is 94,51% when both meta and walking features are jointly used. In the case where only walking features are used, the baseline and MICE + SVR models have a relative error of 95,05% respectively.

### Recursive feature elimination (RFE)

**Fig. 1:**
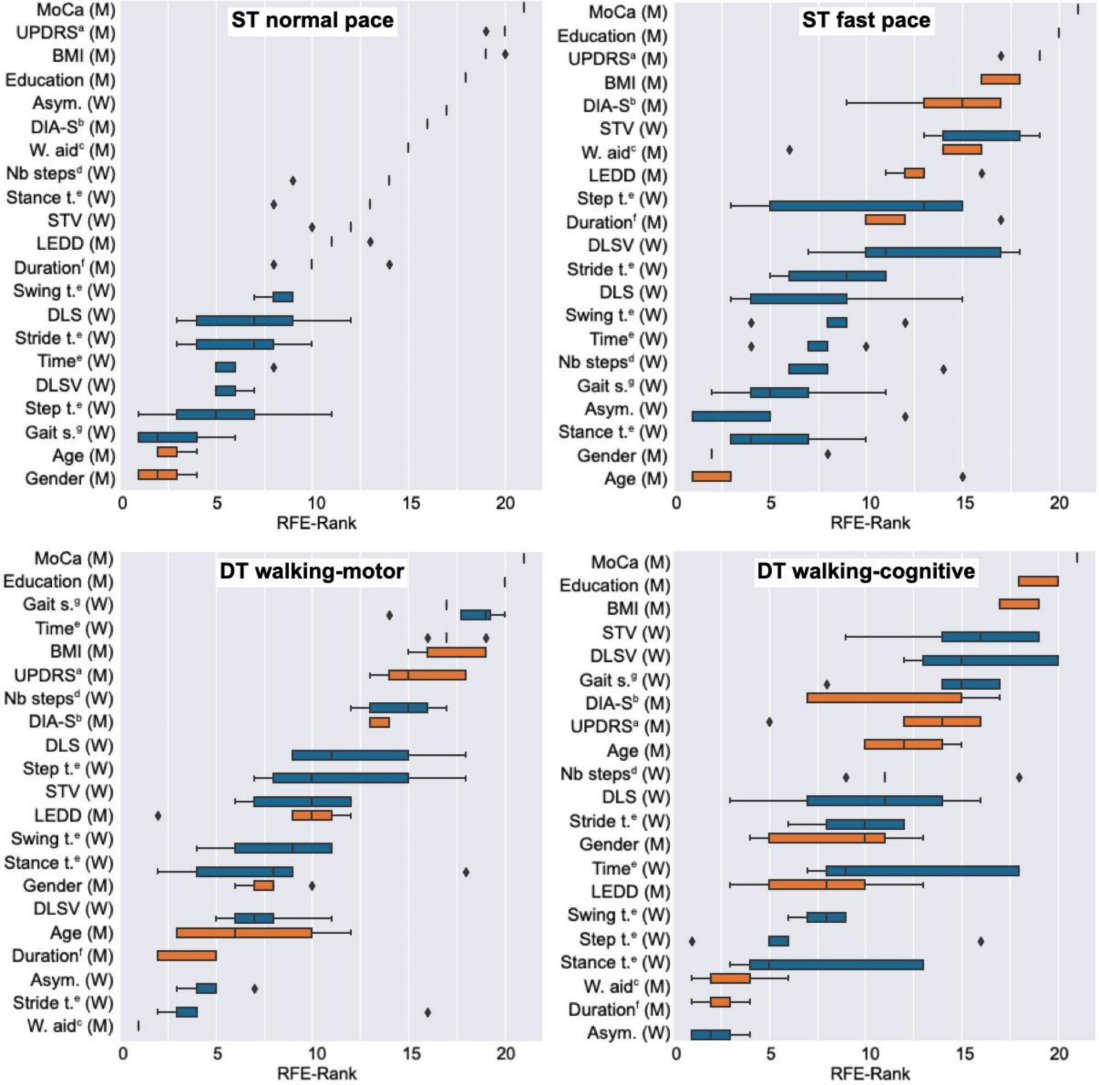
Boxplot for each walking condition: top left ST normal, top right ST fast, bottom left DT walking-motor, and bottom right DT walking-cognitive. The boxplots show when a particular feature was dropped using RFE. Both meta features (M, orange) and walking features (W, blue) were used. All RFE-values vary between 1 (least important feature, therefore dropped first) and 21 (most important feature) and are provided as the average of 5 independent RFE runs. Abbreviations: ^a^ UPDRS-III total score, ^b^ DIA-S total score, ^c^ Walking aid usage in percent, ^d^ Number of steps, ^e^ Time in seconds, ^f^ Disease duration in years, ^g^ Gait speed in meters per second.

First, we performed RFE to analyze the importance of different features, including meta features and walking features. Figure 1 shows the progression of feature elimination using RFE, indicating when each specific feature was dropped. The RFE-Rank represents the feature importance for our machine learning model. All values vary from 1 (least important feature, therefore dropped first) to 21 (most important feature) and are provided as the average of 5 independent RFE runs. Our analysis demonstrates that a combination of meta and walking features yields the best performance in our model. In all four walking conditions, the MoCA score is the single most important predictor for Δ-TMT, followed by other meta features such as education or UPDRS III. Note that there is some variance in the rank of each feature, which is due to the fact that RFE is non-deterministic and was applied multiple times. However, for the ST normal condition, this variability is relatively low, indicating consistent ranking of feature importance. In contrast, the other three walking conditions exhibit a lower level of consistency in their rankings.

**Fig. 2:**
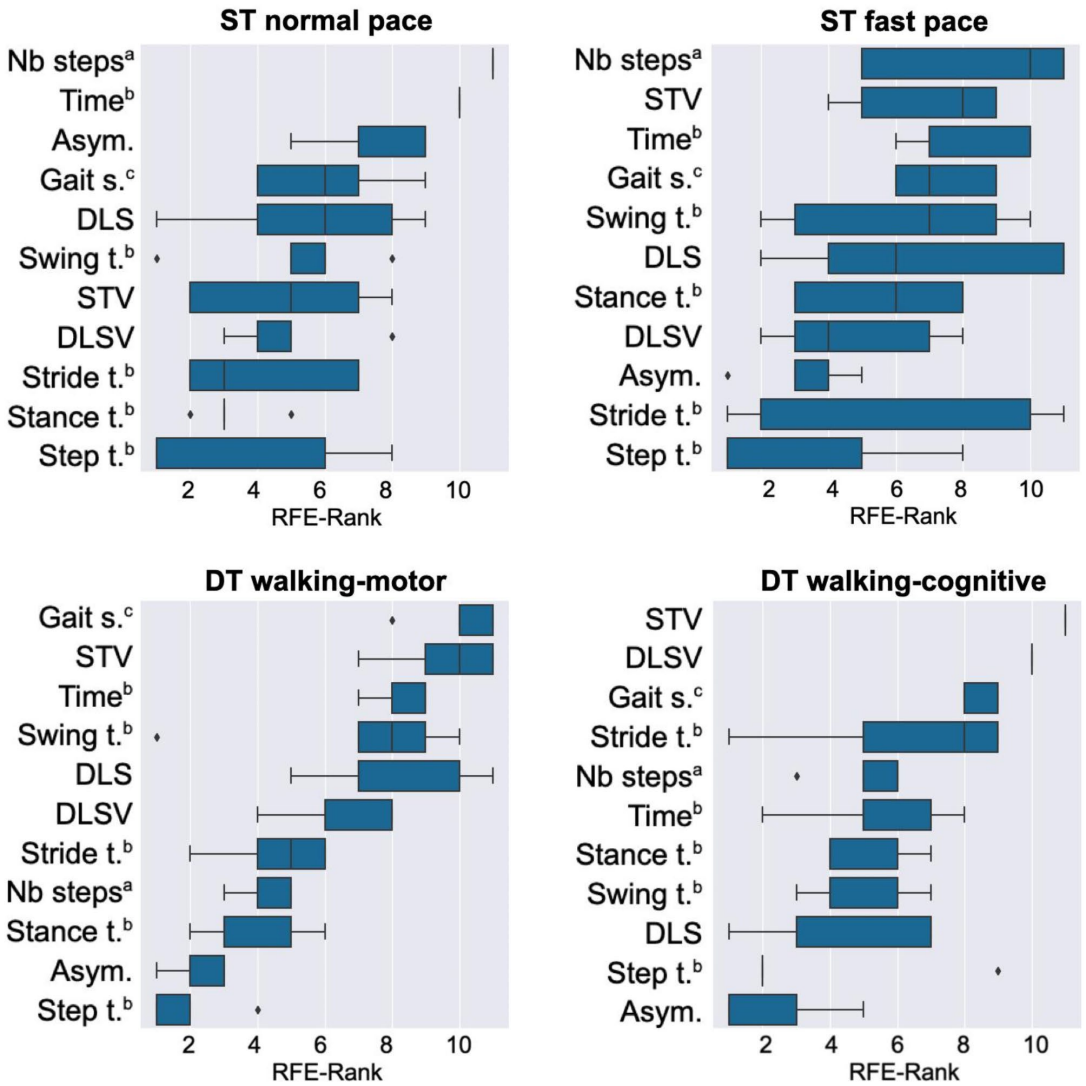
Boxplot for each walking condition: top left ST normal, top right ST fast, bottom left DT walking-motor, and bottom right DT walking-cognitive. The boxplots show when a particular feature was dropped using RFE, when using walking features only. All RFE-values vary between 1 (least important feature, therefore dropped first) and 11 (most important feature) and are provided as the average of 5 independent RFE runs. Abbreviations: ^a^ Number of steps, ^b^ Time in seconds, ^c^ Gait speed in meters per second.

Second, we conducted another analysis, for each walking condition separately, but this time only including walking features, as shown in Fig. 2. Since our initial analysis revealed a significant relationship between walking features and Δ-TMT only in the DT walking-cognitive condition (Table 3), here we specifically focus on this condition (Fig. 2, lower right plot). Our analysis shows that step time variability (STV), double limb support time variability (DLSV) and gait speed emerged as the most relevant features, followed by stride time and number of steps. Among the first three features there was almost no variability in the importance rankings although RFE was run multiple times, thus indicating a robust effect.

## Discussion

We investigated the relationship between gait characteristics and executive functioning in patients with Parkinson’s disease using machine learning approaches. Our analyses were based on a dataset of 103 geriatric patients with Parkinson’s disease who performed different walking conditions with varying levels of difficulty. We employed five imputation methods and four regression approaches to predict executive functioning measured by the Trail-Making Test (TMT). Our findings show that the SVR with MICE imputation reduced the MAE by 4.95% compared to baseline for the best walking condition (i.e. DT walking-cognitive) when only walking features are used, as shown in Figure S3. They also revealed that walking features were significantly related to Δ-TMT, but only in the dual task condition with cognitive demands. Here, step time variability, double limb support time variability and gait speed were among the most prominent features to predict Δ-TMT. Taken together, these findings help to disentangle the complex relationship between motor abilities and executive functioning in Parkinson’s disease, and they further highlight the potential of machine learning methods in studying the underlying principles of movement disorders.

Our first analysis included both meta and walking features (Fig. 1). It revealed that meta features, especially MoCA and education, outperformed most of the walking features with regard to their predictive value on Δ-TMT (i.e. executive functioning). This is not surprising, and confirms our methodological approach, given that MoCA quantifies different cognitive domains, including visuospatial/executive abilities, language and verbal abilities, memory as well as attention^22,23^. Age, which typically correlates with cognitive abilities^46^, was among the features with low predictive value in both single task conditions and the dual task condition with motor demands (Fig. 1, lower left). However, in the dual task condition with cognitive demands (Fig. 1, lower right) age slightly moved up to one of the middle positions.

As our main finding, we can show a significant correlation between walking features and Δ-TMT (i.e. executive functioning) only in the dual task condition with cognitive demands (Table 3). Importantly, the second RFE analysis that only included walking features (Fig. 2) revealed step time variability, double limb support time variability, and gait speed as the most important predictors. As such, our findings help to explain previous, partly contradictory, findings by showing that not only one but a combination of several spatio-temporal walking features can relate to cognitive abilities^3,5,12–15^. In fact, the association of executive functioning and walking features, such as step time variability in complex walking conditions, was investigated before^12^, and a previous study with hospitalized Parkinson’s disease patients indicates that performance in the TMT is not a significant linear predictor for single spatio-temporal walking features^13^. Thus, our more holistic perspective on a patient’s walking profile provides a much better approach to support the conclusion of a relationship between executive functioning and walking features. From a broader view, it demonstrates that machine learning offers a powerful way to more systematically analyze complex data in the context of Parkinson’s disease^19^.

Our results also support the hypothesis that in patients with Parkinson’s disease an additional attentional demand (as under the DT walking-cognitive condition) has a negative influence on their walking profile. In this condition, patients tend to walk more slowly, require more steps, and tend to have higher step time variability (Table S1). These aspects can be interpreted as indicators of insecure, more cautious walking behavior and are associated with an increased risk of falls and greater disease severity^47^. Reduced walking speed and higher gait variability under additional cognitive demand has been described for both healthy individuals and patients with Parkinson’s disease^2,48^. As an explanatory approach for this phenomenon, an interconnection between automated motor processes and higher-order cognitive processes in terms of motor control through executive functioning is suggested^49^. Thus, under ST (i.e., in situations where movement sequences can be more automated under standardized examination conditions because distractors are eliminated), global cognitive performance and other meta features predict Δ-TMT rather than walking features. In addition, the complexity of the demand seems to play a role, as can be seen in the descriptive comparison between the two dual task conditions (walking condition with convergent, i.e. motor, secondary task vs. walking condition with divergent, i.e. cognitive, secondary task). In the clinical context, it is crucial to understand these underlying mechanisms of disease-related limitations, and a better understanding of predictive factors for executive functioning can contribute to the improvement of patient-oriented diagnostics and therapy. For example, these analyses can be used to identify and prevent potentially risky situations (as shown here, e.g. DT walking situations with additional cognitive demands).

Finally, our work has several strengths but also potential limitations, which both could guide future research. First, the order of our four conditions was not random but predetermined due to the increasing task demands and limited motor abilities of the patients. This led to an imbalance in missing data, especially in the DT conditions, which might have a negative impact on the regression analysis. However, the DT walking condition with additional cognitive demand, which was predictive of executive functioning, did not exhibit more missing values than the two single task conditions. Second, we not only tested one but several imputation methods in combination with different ML approaches. While the combination of MICE and SVR performed best on this specific dataset (Figure S2 and S3), others performed comparably, which further emphasizes the strength and robustness of machine learning in the context of complex medical data. Third, our findings are based on a rather large sample but the results may not be generalizable since our cohort of Parkinson’s disease patients was highly selective. In fact, they were hospitalized, of rather high age and some patients were cognitively impaired. Fourth, although well established the TMT is just one proxy for executive functioning and more specific tests may help to further tap into the different domains of executive functioning^30^. Specifically, these are the Verbal Fluency Test (VFT), Clock Drawing Test, Digits Forward and Backward tests, Stroop Test, and Wisconsin Card Sorting Test. The TMT, however, can easily be administered in the clinical context, has good psychometric properties, and therefore allows a comprehensive assessment of cognitive flexibility, processing speed, and task-switching abilities. Fifth, while meta features were only weakly correlated with other meta or walking features, walking features showed a stronger interrelationship (Table S3), which indicates redundancy in the chosen feature representation. Finally, the relative MAE when only walking features are used is 95.05%, which means the combination of MICE with SVR leads to an error 4.95% lower than the baseline, demonstrating that this approach is effective in handling missing data for the prediction of Δ-TMT. Although the significance of such modest improvement depends on the application scenario, in some cases, including the clinical context, even small gains in predictive accuracy can be important.

To conclude, in a cohort of advanced Parkinson’s disease inpatients, step time variability, double limb support time variability, and gait speed were the most important predictors of executive functioning. This relationship was established using a machine learning analysis, only including walking features, and specifically for a dual task walking condition with additional cognitive demands. This adds to our understanding of the complex relationship between cognition and walking abilities in Parkinson’s disease. At the same time, our study provides further evidence for the high potential of machine learning in investigating Parkinson’s disease and possibly other neurodegenerative disorders.

## Electronic supplementary material

Below is the link to the electronic supplementary material.

Supplementary Material 1

## Data Availability

All data and analysis codes are available from the corresponding authors upon reasonable request, including a formal project outline. The patients’ data are not publicly available due to privacy regulations.
