# Supplementary material for: Predicting executive functioning from walking features in Parkinson’s disease using machine learning
Source: Sci Rep. 2024 Nov 27;14:29522. doi: 10.1038/s41598-024-80144-4 (PMC11603322; doi:10.1038/s41598-024-80144-4)

***Supplementary information***

**–**

**Predicting Executive Functioning from Walking Features**

**in Parkinson’s Disease using Machine Learning**

**Running title**: Executive functioning in Parkinson’s disease

Artur Piet^1^, Johanna Geritz^2^, Pascal Garcia^1^, Mona Irsfeld^1^, Frédéric Li^3^, Xinyu Huang^1^, Muhammad Tausif Irshad^1^, Julius Welzel², Clint Hansen², Walter Maetzler², Marcin Grzegorzek^1,4^, and Nico Bunzeck^4^

^1^ Institute of Medical Informatics, University of Luebeck, Germany

^2^ Department of Neurology, University Hospital Schleswig-Holstein, Kiel, Germany

^3^ German Research Center for Artificial Intelligence, Luebeck, Germany

^4^ Department of Psychology and Center of Brain, Behavior and Metabolism (CBBM), University of Luebeck, Germany

**Corresponding authors**

*Artur Piet*

Institute of Medical Informatics, University of Luebeck, Germany, Ratzeburger Allee 160, 23562 Lübeck; email: [ar.piet@uni-luebeck.de](mailto:ar.piet@uni-luebeck.de)

*Nico Bunzeck*

Department of Psychology, University of Luebeck, Maria-Goeppert-Strasse 9a, 23562 Luebeck, Germany; email: [nico.bunzeck@uni-luebeck.de](mailto:nico.bunzeck@uni-luebeck.de), phone: +49 451 3101 3600

**Table S1:** Information on walking features for all four walking conditions.

| **Walking feature** | **ST normal** | **ST fast** | **DT walking-motor** | **DT walking-cognitive** | **Missingness** |
| --- | --- | --- | --- | --- | --- |
| Steps [x/20 m]  Gait speed [m/s]  Time [s for walking 20 m]  Step time [s]  Stride time [s]  Stance time [s]  Swing time [s]  Asymmetry ^a^ [s]  STV [s]  DLS [s]  DLSV [s] | 38.8  0.77  28.3  0.46  0.89  0.78  0.12  0.023  0.010  0.34  0.004 | 36.0  0.95  23.4  0.51  1.01  0.85  0.13  0.033  0.045  0.38  0.038 | 43.6  0.72  31.4  0.49  0.98  0.85  0.13  0.034  0.034  0.36  0.025 | 40.4  0.71  30.7  0.61  0.99  1.12  0.16  0.033  0.048  0.46  0.039 | 18.2%  17.2%  17.8%  17.8%  18.1%  16.9%  17.5%  18.1%  18.4%  17.8%  18.4% |

Abbreviations: DLS, double limb support in seconds [s]; DLSV, double limb support variability; DT, dual task; ST, single task ; STV, step time variability.

**Table S2:** Optimal sets of features selected by RFE for all four walking conditions, in both configurations using meta + walking features, and walking features only. The features are ordered from least to most useful.

| Walking condition | Meta and walking features selected by RFE |
| --- | --- |
| ST normal | Asymmetry, Education, UPDRS III, BMI, MoCa |
| ST fast | BMI, UPDRS III, Education, MoCa |
| DT walking-motor | Gait speed, UPDRS III, BMI, Education, MoCa |
| DT walking-cognitive | Age, UPDRS III, Gait speed, Time, BMI, Education, MoCa |
| Walking condition | **Walking features selected by RFE** |
| ST normal | Stance time, Swing time, Stride time, DLS, Asymmetry, Time, Steps |
| ST fast | Step time, STV, Swing time, DLS |
| DT walking-motor | DLS, Gait speed |
| DT walking-cognitive | Swing time, Stance time, Time, Steps, DLS, Stride time, Gait speed, DLSV, STV |

Abbreviations: BMI, Body Mass Index; DLS, double limb support; DLSV, double limb support variability; DT, dual task; LEDD, levodopa equivalence daily dose (in milligram, mg); MoCA, Montreal Cognitive Assessment total score; Nb steps, number of steps; ST; single task; STV, step time variability; t., time; UPDRS III, Movement Disorder Society-revised version of the motor part of the Unified Parkinson's Disease Rating Scale.


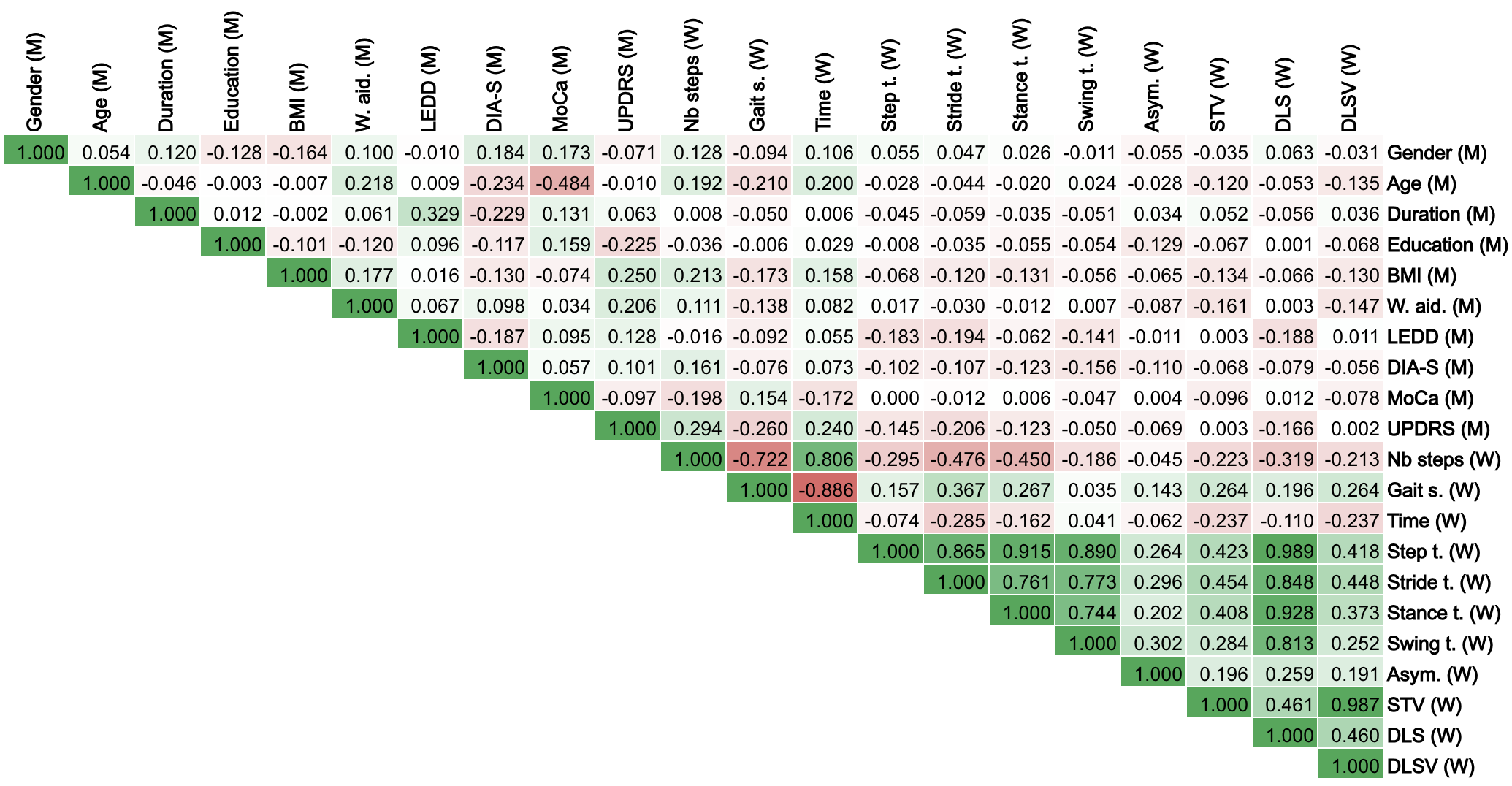


**Table S3**. Correlation matrix of all variables of interest. Values and color code indicate the r-value.

Abbreviations: Asym., asymmetry; BMI, Body Mass Index; DIA-S, *Depression im Alter* Scale, DLS, double limb support; DLSV, double limb support variability; gait s., gait speed; LEDD, levodopa equivalence daily dose (in milligram, mg); M, meta feature; MoCA, Montreal Cognitive Assessment total score; Nb steps, number of steps; STV, step time variability; t., time; UPDRS III, Movement Disorder Society-revised version of the motor part of the Unified Parkinson's Disease Rating Scale; W, walking feature; W.aid, walking aid usage in percentage.


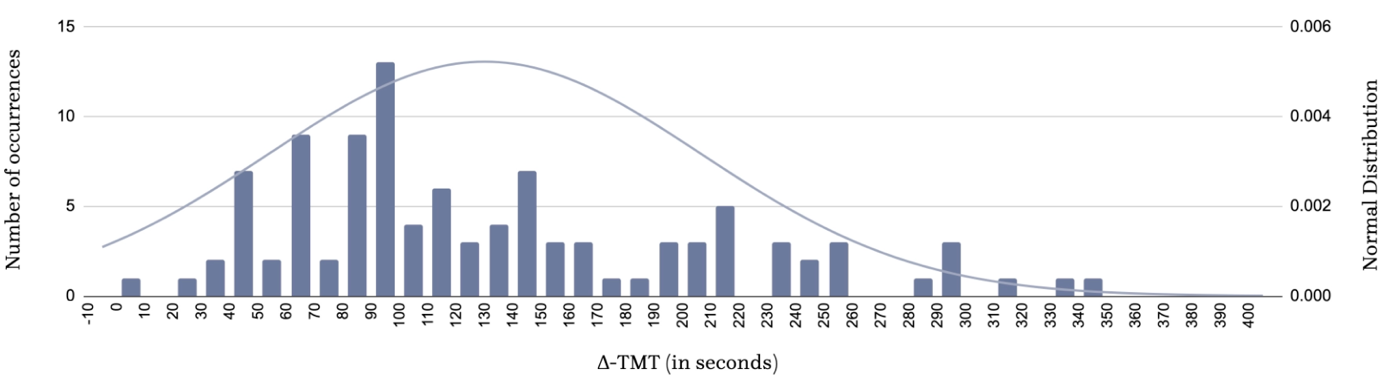


**Figure S1:** Histogram of the Δ-TMT score distribution in the ComOn dataset, sorted in intervals of 10 seconds.

**Figure S2:** Heatmaps showing MAEs for all four walking conditions: ST normal top left, ST fast top right, DT motor bottom left, and DT cognitive bottom right. Each subplot shows prediction results of all combinations of imputation (baseline, i.e. mean imputation, kNN, MICE, MF, and MIDAS) and regression (RF, XGB, SVR, and MLP). All features (meta and walking) were used. The color corresponds to the MAE in blue (darker are lower errors).


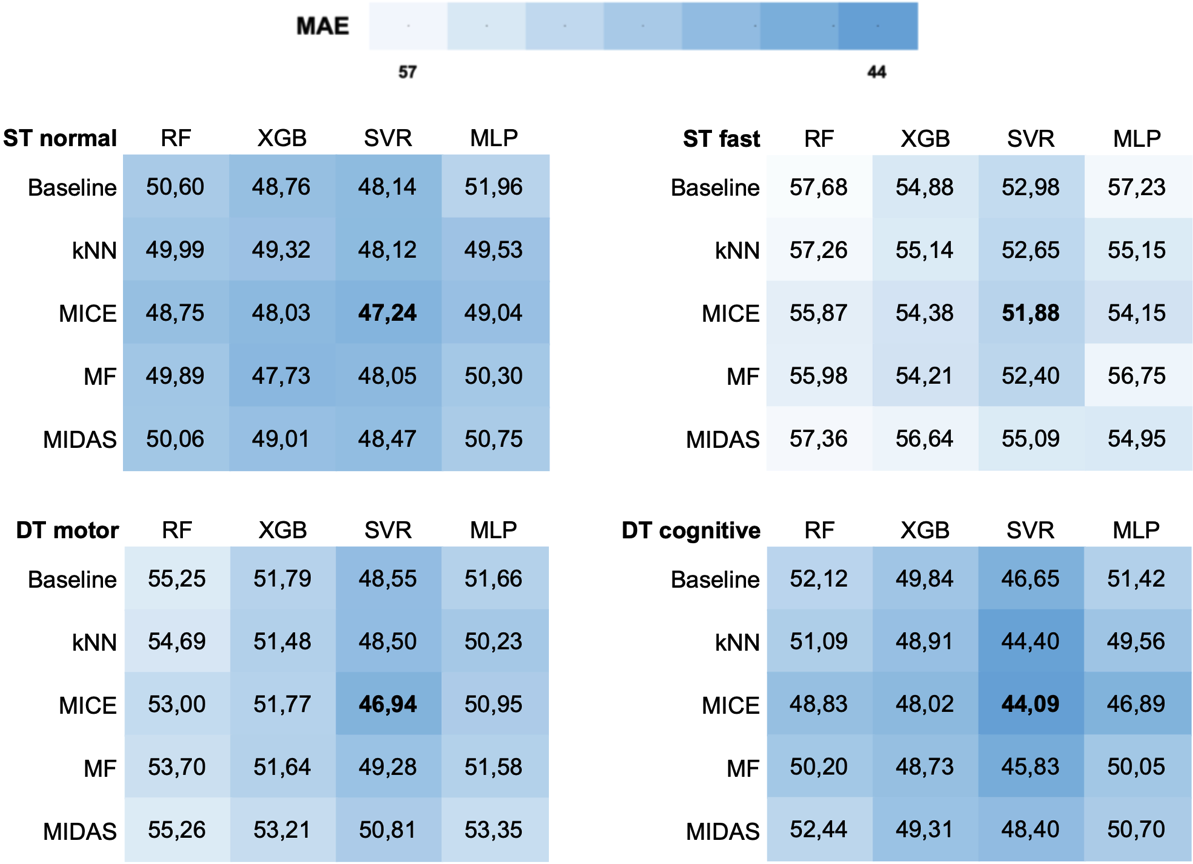

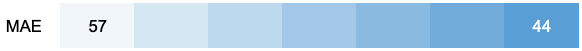


**Figure S3:** Heatmaps showing MAEs all four walking conditions: ST normal top left, ST fast top right, DT motor bottom left, and DT cognitive bottom right. Each subplot shows prediction results of all combinations of imputation (baseline, i.e. mean imputation, kNN, MICE, MF, and MIDAS) and regression (RF, XGB, SVR, and MLP). Only the walking features were used. The color corresponds to the MAE in blue (darker are lower errors).


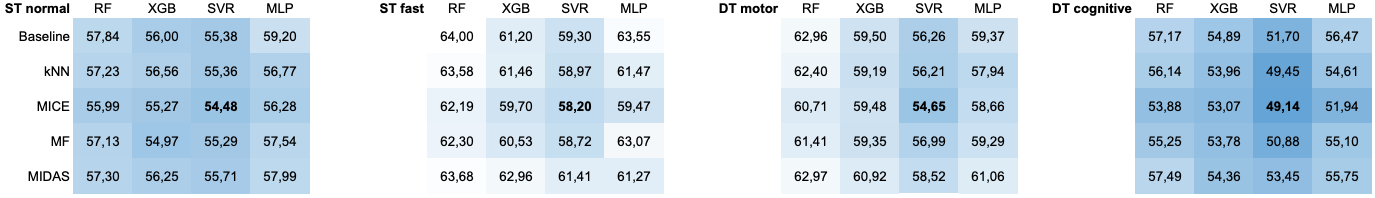

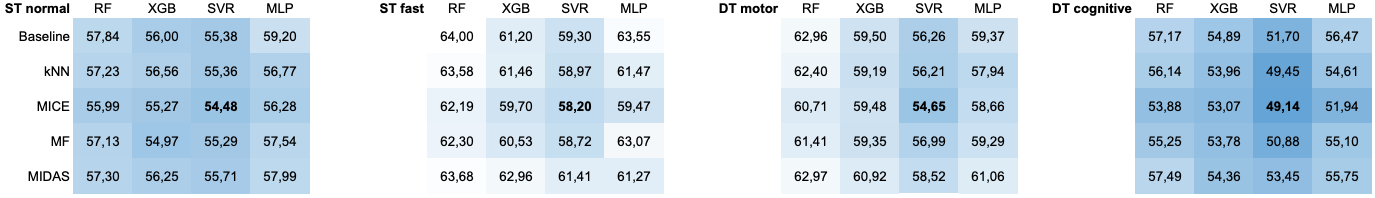

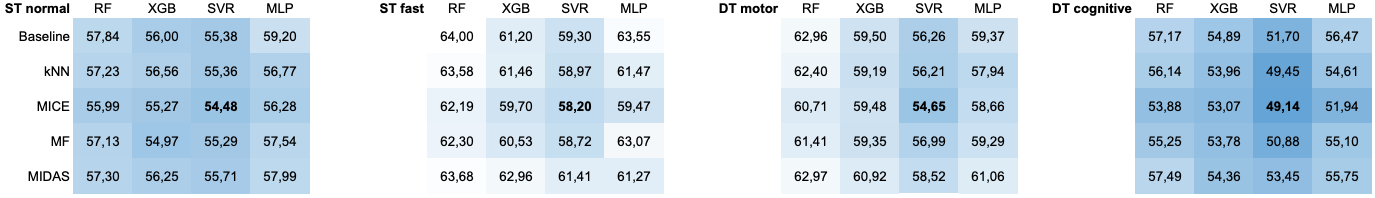

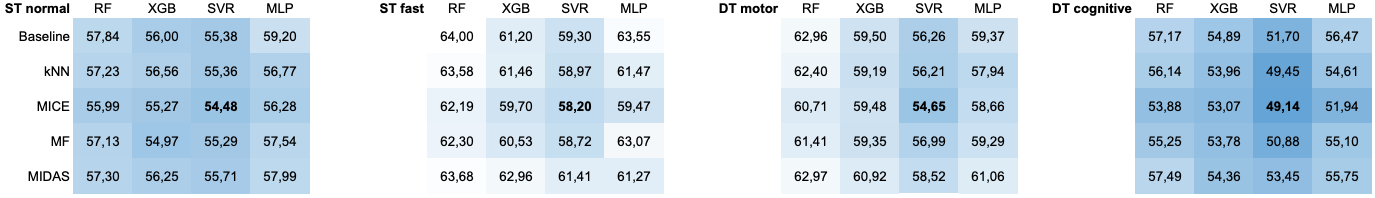

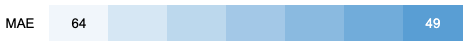

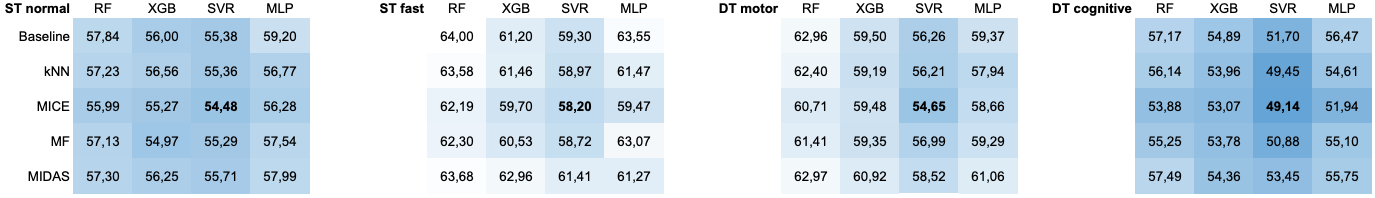

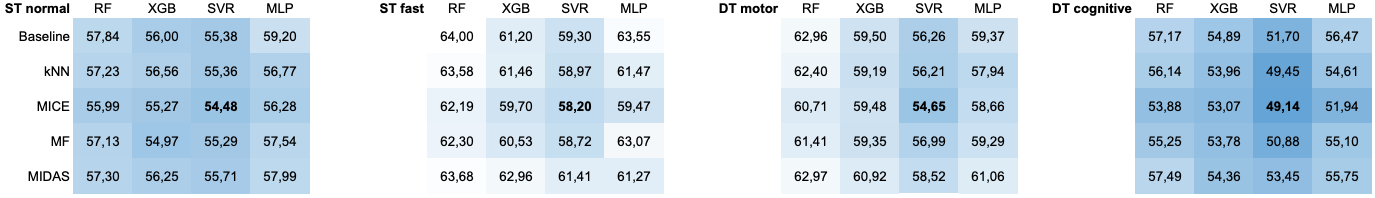

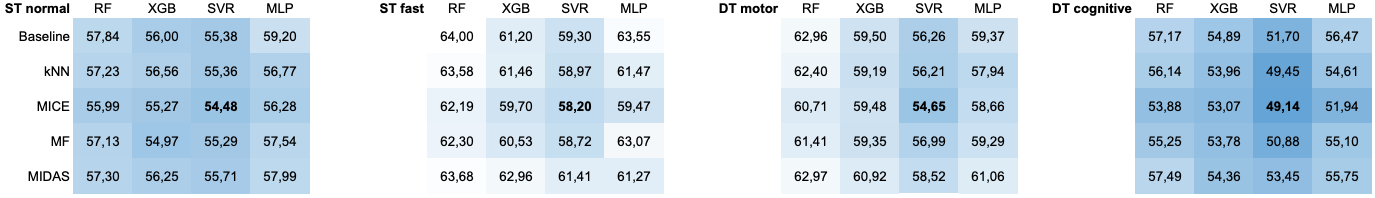


**Figure S4:** Regression evaluation metrics as a function of the number of features selected by RFE. All meta and walking features have been used. The left x-axis indicates values for MAE (blue) and RMSE (black), while the right x-axis shows correlation values (green).


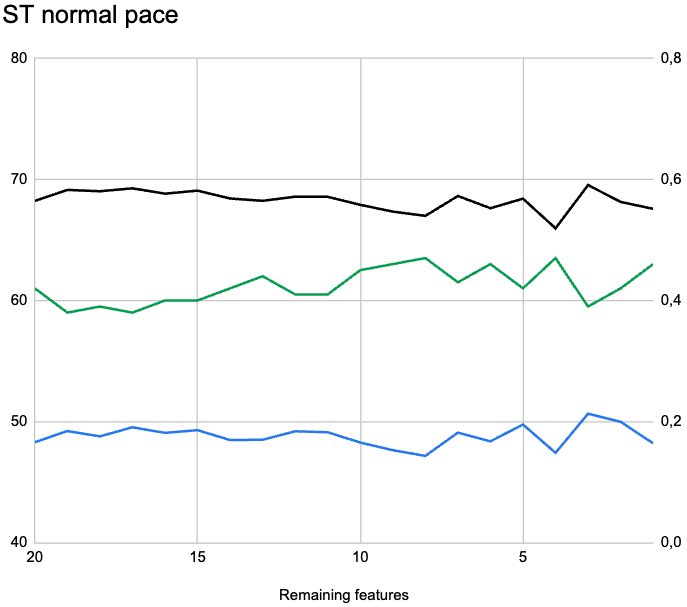

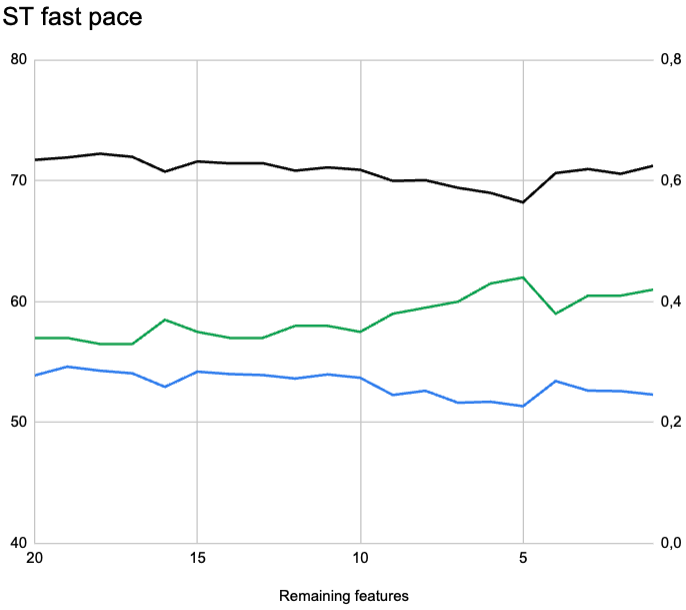

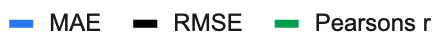


MAE and RMSE

Pearsons r


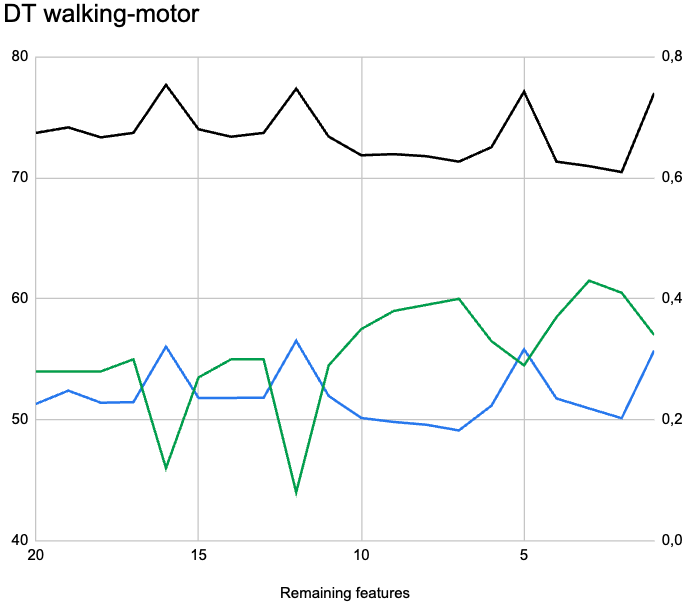

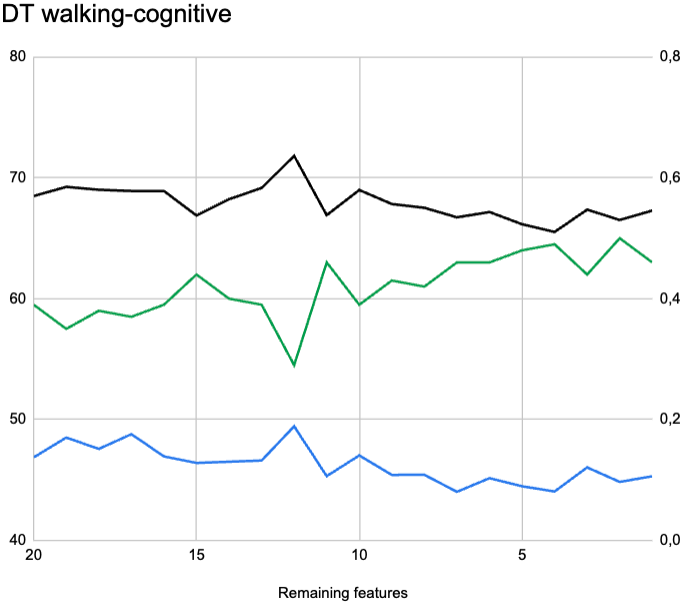


MAE and RMSE

Pearsons r

Remaining features

Remaining features


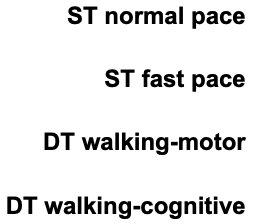

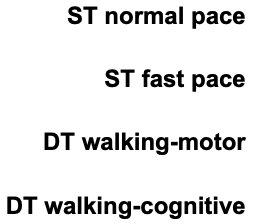

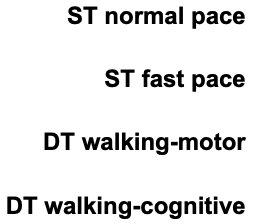

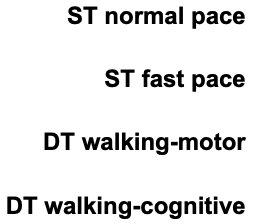

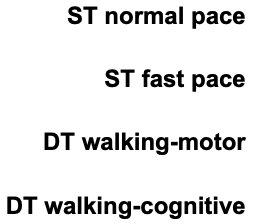

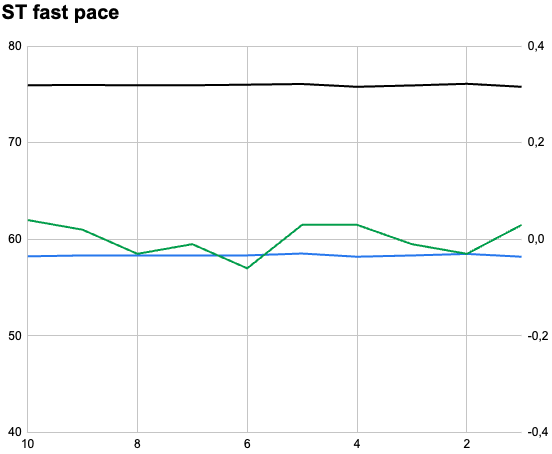


**Figure S5:** Regression evaluation metrics as a function of the number of features selected by RFE. Only the walking features have been used. The left x-axis indicates values for MAE (blue) and RMSE (black), while the right x-axis shows correlation values (green).


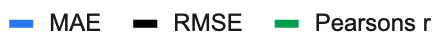


MAE and RMSE

Pearsons r

Remaining features

Remaining features


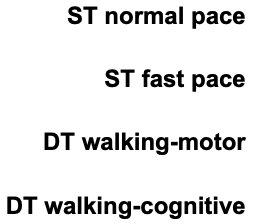

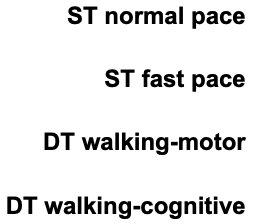

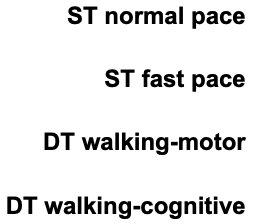


MAE and RMSE


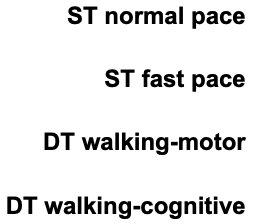

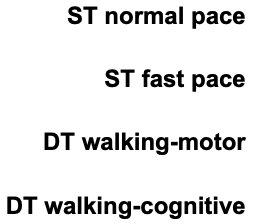


Pearsons r


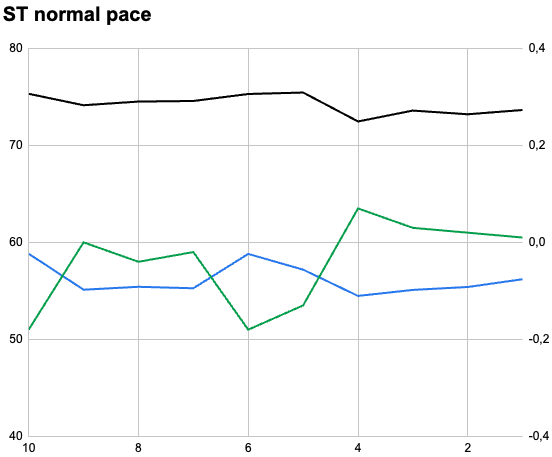

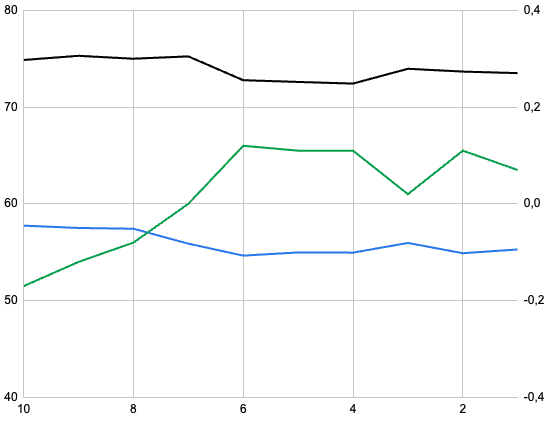

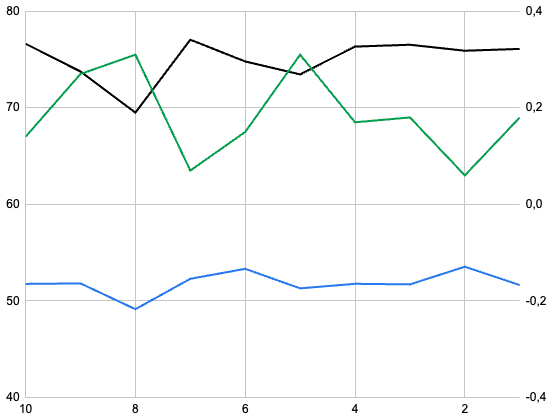


**Figure S6:** Scatter plots of the actual Δ-TMT scores and the scores predicted with MICE imputation and SVR regression using meta and walking features. The linear trendline is shown in blue color.


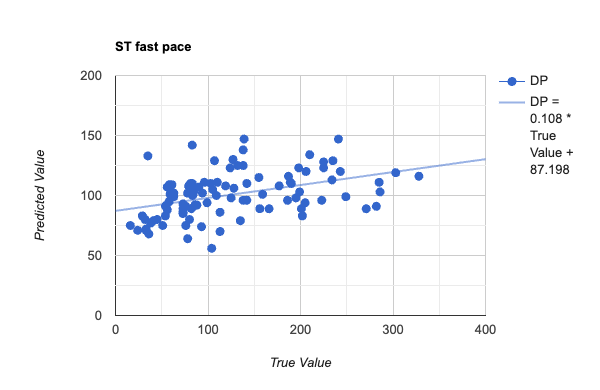

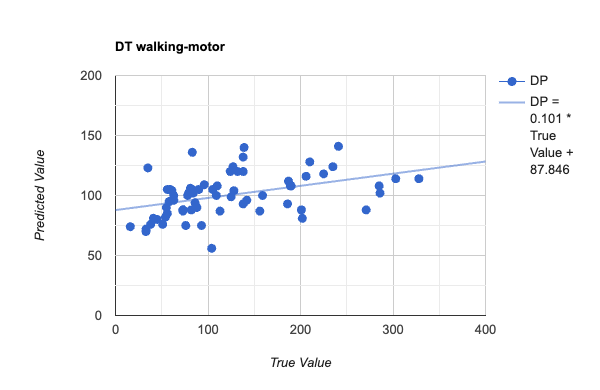

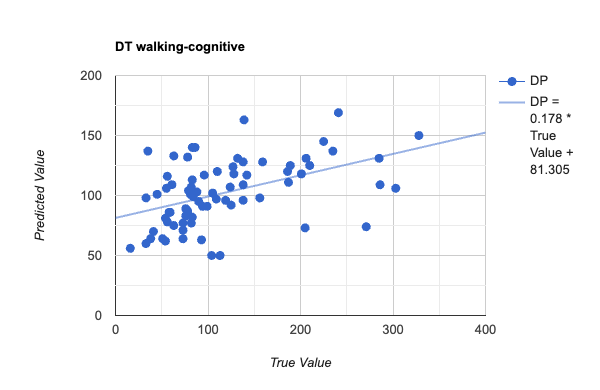

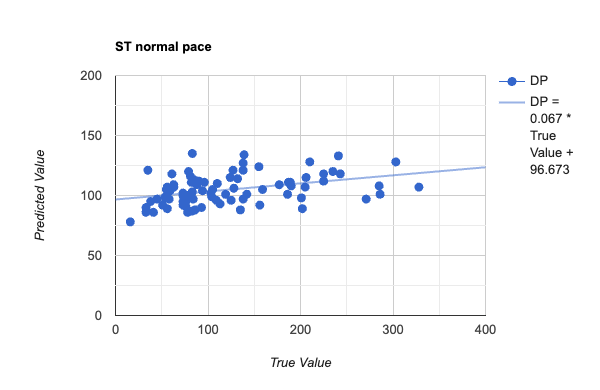

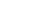

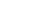

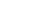

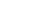

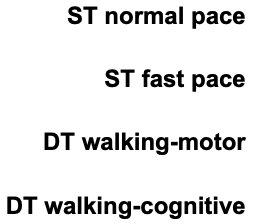

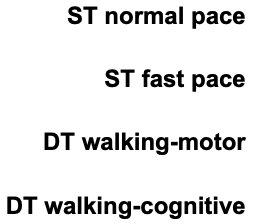

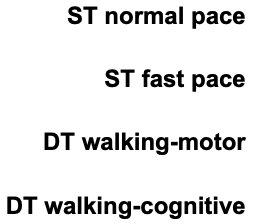

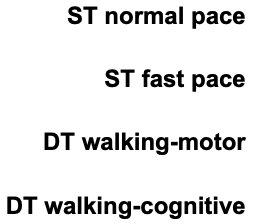

Supplement: Supplementary file 1 — Supplementary Material 1 [file 41598_2024_80144_MOESM1_ESM.docx]
